# Supplementary figures and images for: Type VI secretion system killing by commensal Neisseria is influenced by expression of type four pili
Source: eLife. 2021 Jul 7;10:e63755. doi: 10.7554/eLife.63755 (PMC8263058; doi:10.7554/eLife.63755)

**Figure 1 - Source data 1: Western Blot of *N. cinerea* Hcp secretion and expression**


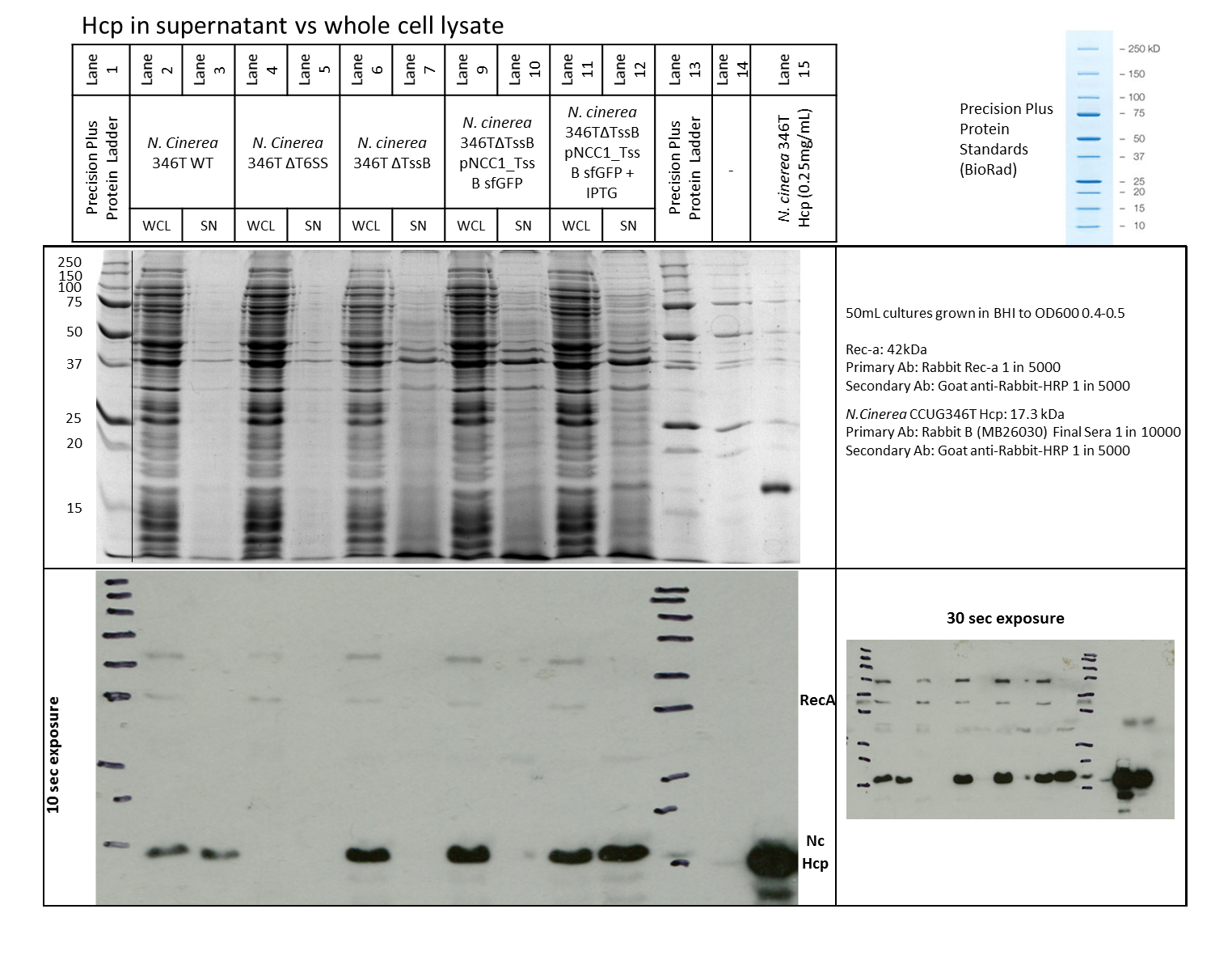

Supplement: Figure 1—source data 1. [file elife-63755-fig1-data1.docx]
