# Supplementary material for: Type VI secretion system killing by commensal Neisseria is influenced by expression of type four pili
Source: eLife. 2021 Jul 7;10:e63755. doi: 10.7554/eLife.63755 (PMC8263058; doi:10.7554/eLife.63755)
Supplement: Supplementary file 1. [file elife-63755-supp1.docx]

**Supplementary file 1. Putative T6SS core components in *N. cinerea* 346T.**

| **Name** | **Protein Size (aa)** | **% aa (coverage) identity with *P. aeruginosa* PAO1** | **COG** | **Putative Localisation** | **Predicted Function** |
| --- | --- | --- | --- | --- | --- |
| **TssJ** | 215 | 35 (58) | COG3521 | Outer membrane | Membrane complex |
| **TssL** | 421 | 36 (91) | COG3455 | Inner membrane | Membrane complex |
| **TssM** | 1185 | 27 (93) | COG3523 | Inner membrane | Membrane complex |
| **TssK** | 447 | 37 (99) | COG3522 | Inner membrane | Baseplate complex |
| **TssF** | 640 | 33 (99) | COG3515 | Inner membrane | Baseplate complex |
| **TssG** | 346 | 37 (92) | COG3520 | Inner membrane | Baseplate complex |
| **TssE** | 170 | 37 (97) | COG3518 | Cytoplasmic | Baseplate complex |
| **TssA** | 355 | 28 (98) | COG3515 | Cytoplasmic | Tail complex |
| **TssB** | 172 | 70 (98) | COG3157 | Cytoplasmic | Tail complex (sheath) |
| **TssC** | 499 | 73 (97) | COG3517 | Cytoplasmic | Tail complex (sheath) |
| **ClpV** | 883 | 59 (98) | COG0542 | Cytoplasmic | ATPase |
| **Hcp** | 160 | 41 (100) | COG3157 | Cytoplasmic/Inner membrane | Tail complex (Hcp tube) |
| **VgrG** | 757 | 35 (99) | COG3501 | Inner membrane | Spike |
