# Supplementary material for: Type VI secretion system killing by commensal Neisseria is influenced by expression of type four pili
Source: eLife. 2021 Jul 7;10:e63755. doi: 10.7554/eLife.63755 (PMC8263058; doi:10.7554/eLife.63755)
Supplement: Transparent reporting form [file elife-63755-transrepform.docx]

***eLife’s* transparent reporting form**

We encourage authors to provide detailed information *within their submission* to facilitate the interpretation and replication of experiments. Authors can upload supporting documentation to indicate the use of appropriate reporting guidelines for health-related research (see [EQUATOR Network](http://www.equator-network.org/%20)), life science research (see the [BioSharing Information Resource](https://biosharing.org/)), or the [ARRIVE guidelines](http://www.plosbiology.org/article/info:doi/10.1371/journal.pbio.1000412) for reporting work involving animal research. Where applicable, authors should refer to any relevant reporting standards documents in this form.

If you have any questions, please consult our Journal Policies and/or contact us: [editorial@elifesciences.org](mailto:editorial@elifesciences.org).

**Sample-size estimation**

- You should state whether an appropriate sample size was computed when the study was being designed
- You should state the statistical method of sample size computation and any required assumptions
- If no explicit power analysis was used, you should describe how you decided what sample (replicate) size (number) to use

Please outline where this information can be found within the submission (e.g., sections or figure legends), or explain why this information doesn’t apply to your submission:

No explicit power analysis was used. The number of replicates chosen was based on our standard laboratory procedures and our experience and expertise in bacterial growth. The number of independent experiments/biological repeats (replicates) is stated in the Figure legends and Methods sections of the paper; generally this was at least two biological replicates (independent experiments).

**Replicates**

- You should report how often each experiment was performed
- You should include a definition of biological versus technical replication
- The data obtained should be provided and sufficient information should be provided to indicate the number of independent biological and/or technical replicates
- If you encountered any outliers, you should describe how these were handled
- Criteria for exclusion/inclusion of data should be clearly stated
- High-throughput sequence data should be uploaded before submission, with a private link for reviewers provided (these are available from both GEO and ArrayExpress)

The number of times the experiment was performed (independent experiment or biological repeat) and, where relevant, the number of images analysed, is stated in the Methods, Figure legends and Figure supplement legends. The number of cells analysed in fluorescence microscopy (Figure 2C) is indicated in the figure.

Each independent experiment is a biological replicate performed on a different day using independently prepared bacterial cultures. Where applicable figures contain individual data points representing each technical replicate from each independent experiment.

Reasons for excluding data from Flow cytometry experiments is outlined in the legend to figure 7 supplement 2 and for excluding CFU counts from single spot (where colonies were merged an unable to be counted) is included in the source data file 1 for Figure 7.

Whole genome sequence (PacBio) data has been uploaded to Dryad.

Please outline where this information can be found within the submission (e.g., sections or figure legends), or explain why this information doesn’t apply to your submission:

**Statistical reporting**

- Statistical analysis methods should be described and justified
- Raw data should be presented in figures whenever informative to do so (typically when N per group is less than 10)
- For each experiment, you should identify the statistical tests used, exact values of N, definitions of center, methods of multiple test correction, and dispersion and precision measures (e.g., mean, median, SD, SEM, confidence intervals; and, for the major substantive results, a measure of effect size (e.g., Pearson's r, Cohen's d)
- Report exact p-values wherever possible alongside the summary statistics and 95% confidence intervals. These should be reported for all key questions and not only when the p-value is less than 0.05.

Please outline where this information can be found within the submission (e.g., sections or figure legends), or explain why this information doesn’t apply to your submission:

Statistical methods, number of replicates, means, standard deviation and p-values are shown in the Figures and respective Figure legends where appropriate. The statistical methods are described in the Methods section of the manuscript.

(For large datasets, or papers with a very large number of statistical tests, you may upload a single table file with tests, Ns, etc., with reference to sections in the manuscript.)

**Group allocation**

- Indicate how samples were allocated into experimental groups (in the case of clinical studies, please specify allocation to treatment method); if randomization was used, please also state if restricted randomization was applied
- Indicate if masking was used during group allocation, data collection and/or data analysis

Please outline where this information can be found within the submission (e.g., sections or figure legends), or explain why this information doesn’t apply to your submission:

No group allocation or masking was applied.

**Additional data files (“source data”)**

- We encourage you to upload relevant additional data files, such as numerical data that are represented as a graph in a figure, or as a summary table
- Where provided, these should be in the most useful format, and they can be uploaded as “Source data” files linked to a main figure or table
- Include model definition files including the full list of parameters used
- Include code used for data analysis (e.g., R, MatLab)
- Avoid stating that data files are “available upon request”

Please indicate the figures or tables for which source data files have been provided:

Source data files are available for the following figures: Figure 1C and 1D, 2C, 5A-F, 6A-C, 7D and 8A. Source data files are available for the following figure supplements: Figure 2 supplement 1, Figure 5 supplement 1, Figure 7 supplement 1, Figure 7 supplement 2.

For Figure 1A and B- source data is WGS data available on Dryad.
